# Correction: Efficacy and safety assessment of different dosage of benznidazol for the treatment of Chagas disease in chronic phase in adults (MULTIBENZ study): study protocol for a multicenter randomized Phase II non-inferiority clinical trial

**DOI:** 10.1186/s13063-023-07659-5

**Published:** 2023-11-14

**Authors:** D. Molina-Morant, M. L. Fernández, P. Bosch-Nicolau, E. Sulleiro, M. Bangher, F. Salvador, A. Sanchez-Montalva, A. L. P. Ribeiro, A. M. B. de Paula, S. Eloi, R. Correa-Oliveira, J. C. Villar, S. Sosa-Estani, I. Molina

**Affiliations:** 1 Infectious Diseases Department, Vall d’Hebron University Hospital, PROSICS Barcelona, Universitat Autònoma de Barcelona, P° Vall d’Hebron 119, Edifici Mediterrània, VHIR, 08035 Barcelona, Spain; 2 Departamento de Clínica, Patología y Tratamiento, Instituto Nacional de Parasitología Dr. Mario Fatala Chaben, Ministerio de Salud y Desarrollo Social, Buenos Aires, Argentina; 3 Microbiology Department, Vall d’Hebron University Hospital, PROSICS Barcelona, Universitat Autònoma de Barcelona, Barcelona, Spain; 4 Instituto de Cardiología de Corrientes Juana Francisca Cabral (Argentina), Corrientes, Argentina; 5 Programa de Pós-Graduação Infectologia E Medicina Tropical, Faculdade de Medicina da Universidade Federal de Minas Gerais, Belo Horizonte, Minas Gerais Brazil; 6 Laboratory of Health Science, Postgraduate Program in Health Sciences, Universidade Estadual de Montes Claros (Unimontes), Montes Claros, MG Brazil; 7 Programa de Pós-Graduação Em Patologia, Departamento de Propedêutica Complementar, Faculdade de Medicina da Universidade Federal de Minas Gerais, Belo Horizonte, Brazil; 8 Faculdade de Medicina da Universidade José Do Rosário Vellano, Belo Horizonte, Brazil; 9 René Rachou Institute, Oswaldo Cruz Foundation, Belo Horizonte, Brazil; 10 Faculty of Health Sciences, Universidad Autónoma de Bucaramanga and Research Department, Bucaramanga, Colombia; 11 Fundación Cardioinfantil - Instituto de Cardiología, Bogotá, Colombia; 12 Chagas Clinical Program, Drugs for Neglected Disease Initiative (DNDi), Geneva, Switzerland; 13 Epidemiology and Public Health Research Center, CONICET, Buenos Aires, Argentina

**Correction: BMC Trials 21, 328 (2020)**

**https://doi.org/10.1186/s13063-020-4226-2**

Following publication of the original article [1], we have been informed that the MULTIBENZ trial was conceived as a non-inferiority trial, but after the sample size calculation suggested that such a trial would not be feasible, the authors decided to move to a superiority trial. Unfortunately, the protocol version that was used was this old ‘inferiority’ version and not the one that the authors registered at Clinical trials.gov and which they submitted for ethical approval. In summary, the trial was a superiority trial and not a "non-inferiority" trial.

To calculate the new sample size, the authors took into account published data from our research group, which allowed them to maintain a design with an acceptable number of patients.

They considered a superiority design for comparison of proportions. They hypothesized a 50% decrease in the proportion of patients with a positive qPCR or treatment discontinuation due to adverse events during follow-up for the experimental arms compared to the standard treatment arm (300/60d). Based on the previous results of their own group; they estimated a 40% of treatment failures according to the intention-to-treat principle. Given the expected proportions in every group and given that the authors plan two pairwise comparisons (each experimental arm vs standard treatment arm), for a power of 80% and a type I error of 0.05, 237 patients will needed.

The original article has been corrected and track changes are attached to this correction (additional file 1).

### Supplementary Information

**Additional file 1.**
