# Supplementary material for: Correction: Efficacy and safety assessment of different dosage of benznidazol for the treatment of Chagas disease in chronic phase in adults (MULTIBENZ study): study protocol for a multicenter randomized Phase II non-inferiority clinical trial
Source: Trials. 2023 Nov 14;24:726. doi: 10.1186/s13063-023-07659-5 (PMC10644612; doi:10.1186/s13063-023-07659-5)

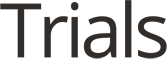
https://doi.org/10.1186/s13063-020-4226-2

STUDY PROTOCOL

Open Access


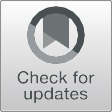
Efficacy and safety assessment of different dosage of benznidazol for the treatment of Chagas disease in chronic phase in adults (MULTIBENZ study): study protocol for a multicenter randomized Phase II superiority clinical trial

D. Molina-Morant^1^, M. L. Fernández^2^, P. Bosch-Nicolau^1^, E. Sulleiro^3^, M. Bangher^4^, F. Salvador^1^,

A. Sanchez-Montalva^1^, A. L. P. Ribeiro^5^, A. M. B. de Paula^6^, S. Eloi^7,8^, R. Correa-Oliveira^9^, J. C. Villar^10,11^,


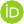
S. Sosa-Estani^12,13^ and I. Molina^1*^

Abstract

Background: Chagas disease (CD) continues to be a neglected infectious disease with one of the largest burdens globally. Despite the modest cure rates in adult chronic patients and its safety profile, benznidazole (BNZ) is still the drug of choice. Its current recommended dose is based on nonrandomized studies, and efficacy and safety of the optimal dose of BNZ have been scarcely analyzed in clinical trials.

Methods/design: MULTIBENZ is a phase II, randomized, superiority, double-blind, multicenter international clinical trial. A total of 240 patients with *Trypanosoma* CD in the chronic phase will be recruited in four different countries (Argentina, Brazil, Colombia, and Spain). Patients will be randomized to receive BNZ 150 mg/day for 60 days, 400 mg/day for 15 days, or 300 mg/day for 60 days (comparator arm). The primary outcome is the efficacy of three different BNZ therapeutic schemes in terms of dose and duration. Efficacy will be assessed according to the proportion of patients with sustained parasitic load suppression in peripheral blood measured by polymerase chain reaction. The secondary outcomes are related to pharmacokinetics and drug tolerability. The follow-up will be 12 months from randomization to end of study participation. Recruitment was started in April 2018.

Conclusion: This is a clinical trial conducted for the assessment of different dose schemes of BNZ compared with the standard treatment regimen for the treatment of CD in the chronic phase. MULTIBENZ may help to clarify which is the most adequate BNZ regimen in terms of efficacy and safety, predicated on sustained parasitic load suppression in peripheral blood.

Trial registration: ClinicalTrials.gov, [NCT03191162](https://clinicaltrials.gov/ct2/show/NCT03191162). Registered on 19 June 2017.

Keywords: Chagas disease, Benznidazole, Therapeutic, Multicenter study, Clinical trial

* Correspondence: [imolina@vhebron.net](mailto:imolina@vhebron.net)

^1^Infectious Diseases Department, Vall d’Hebron University Hospital, PROSICS Barcelona, Universitat Autònoma de Barcelona, P° Vall d’Hebron 119, Edifici Mediterrània, VHIR, 08035 Barcelona, Spain

Full list of author information is available at the end of the article


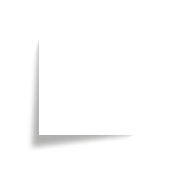

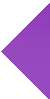


© The Author(s). 2020 Open Access This article is licensed under a Creative Commons Attribution 4.0 International License, which permits use, sharing, adaptation, distribution and reproduction in any medium or format, as long as you give appropriate credit to the original author(s) and the source, provide a link to the Creative Commons licence, and indicate if changes were made. The images or other third party material in this article are included in the article's Creative Commons licence, unless indicated otherwise in a credit line to the material. If material is not included in the article's Creative Commons licence and your intended use is not permitted by statutory regulation or exceeds the permitted use, you will need to obtain permission directly from the copyright holder. To view a copy of this licence, visit <http://creativecommons.org/licenses/by/4.0/>. The Creative Commons Public Domain Dedication waiver (<http://creativecommons.org/publicdomain/zero/1.0/>) applies to the data made available in this article, unless otherwise stated in a credit line to the data.

# Introduction

Chagas disease (CD) is a neglected parasitic infection caused by the protozoan *Trypanosoma cruzi*. It is en- demic in the American continent, and according to the latest estimates, it affects around 6 million people. Thir- teen percent of the Latin American population remains at risk of contracting the infection, which is transmitted to humans by Triatominae insects [[1](#_bookmark3)]. CD has also be- come a rising health problem in nonendemic countries because of international migration, and nonvectorial transmission can occur trhough blood transfusion, organ transplant, and congenital infection [[2](#_bookmark3), [3](#_bookmark3)]. In addition, orally transmitted CD has been detected in endemic areas because of food carrying either infected Triatomi- nae insects or their feces [[4](#_bookmark3)].

After malaria and schistosomiasis, CD represents the third largest parasitic disease burden globally, with more than 15,000 deaths attributed directly to chronic Chagas cardiomyopathy (CCM) annually. CCM is the main complication in the chronic phase of CD, and it develops in approximately 30% of patients chronically infected with *T. cruzi* [[1](#_bookmark3)]. Moreover, it is the most common form of nonischemic cardiomyopathy in Latin America [[5](#_bookmark3), [6](#_bookmark3)].

Currently, there are only two available drugs to treat CD: nifurtimox and benznidazole (BNZ). Of these two, BNZ is the one most studied and most often used as a treatment. How- ever, current schemes of this treatment have some limitations. On the one hand, it has a limited efficacy based on serocon- version (around 50–80% in the acute phase of the disease and 8–20% in the chronic phase) [[7](#_bookmark3)]. Another important limitation is the high rate of adverse events (AEs) when using these drugs. The incidence of AEs related to BNZ varies from 40– 50% up to 98%, and around 15% of these patients have to de- finitively stop the treatment for this reason, with the rate even higher in patients treated with nifurtimox [[8](#_bookmark3)–[10](#_bookmark3)]. The most commonly observed AEs are hypersensitivity (rash, fever, gen- eralized edema, lymphadenopathy, myalgia, and arthralgia), gastrointestinal disorders, bone marrow toxicity (neutropenia and thrombocytopenic purpura), and peripheral polyneurop- athy [[9](#_bookmark3)]. Current knowledge about the BNZ toxicity mecha- nisms is scarce because the main studies have focused on the clinical aspects of these AEs [[10](#_bookmark3)]. Our group recently carried out an analysis of the cytokine profile and human leukocyte antigen (HLA) classes I and II of patients who were treated with BNZ, and we found a higher treatment discontinuation rate due to skin hypersensitivity AEs in patients who had the HLA-B*3505 allele [[11](#_bookmark3)].

Moreover, another drawback of the studies assessing the efficacy of BNZ in chronic CD is the lack of a biomarker to define the cure of disease. Currently, the cure criteria are negative seroconversion of two serologic assays against different antigens, but it usually takes several years after an effective treatment, precluding its use in clinical trials. In addition, detection of *T. cruzi* DNA in peripheral blood

cannot be used to define cure, because a negative result does not mean absence of the infection; however, in recent years, it has become an important tool used to identify therapeutic failure when the result remains positive after completed treatment [[8](#_bookmark3)].

Antitrypanosomal treatment is always recommended for acute and congenital CD, reactivated CD infections, and chronic CD in individuals younger than 18 years of age [[3](#_bookmark3), [12](#_bookmark3)]. Despite the limitations of treatment of chronic CD in adults, international guidelines recommend treatment with either BNZ or nifurtimox in patients under 50 years old with nonestablished cardiac complications [[13](#_bookmark3), [14](#_bookmark3)]. This is based mainly on the lower long-term clinical progression observed in patients treated with BNZ after a mean follow-up of 10 years, the parasite persistence and concomitant chronic in- flammation underlying CCM, and the prevention of vertical transmission to children born by infected women and treated before pregnancy [[3](#_bookmark3), [15](#_bookmark3)]. Results of a systematic re- view and meta-analysis showed little benefit of the treatment, and the BENEFIT (Evaluation of the Use of Antiparasital Drug [Benznidazole] in the Treatment of Chronic Chagas’ Disease) trial found no statistically significant reduction of cardiac clinical impairment in patients with moderate to se- vere cardiomyopathy [[16](#_bookmark3), [17](#_bookmark3)]. Treatment should be individu- alized for patients older than 50 years of age and for patients with comorbidities [[3](#_bookmark3)].

BNZ dosing and duration

Currently, the recommended BNZ dosage and duration regimen for CD treatment is 5–7 mg/kg/day for 60 days. This recommendation is based on studies carried out in the 1970s [[18](#_bookmark3)]. However, nowadays, both the dose and duration of treatment are under discussion, predicated in findings from CD murine models, pharmacokinetic (PK)/pharmacodynamic studies, and studies in patients who discontinued the treatment. On this basis, it seems clear that BNZ dose may be optimized.

*Lower dose*

Two population PK studies have shown through mathem- atical models that lower dosage with the same duration would have the same efficacy [[19](#_bookmark3), [20](#_bookmark3)]. One of them [[19](#_bookmark3)] was carried out in children and the other in adults [[20](#_bookmark3)]. In the pediatric study, children were treated with a standard dose of BNZ. Although significantly lower concentrations of the drug were achieved compared with those reported in adults, the treatment was effective in all patients who completed the treatment course. Moreover, data from a second study carried out in adults revealed that a dose of 5 mg/kg/day might lead to overexposure in the majority of patients and that a BNZ dose of 2.5 mg/kg/day is enough to adequately keep BNZ trough plasma concentrations within the recommended target range according to previ- ous PK studies [[21](#_bookmark3), [22](#_bookmark3)].

*Higher dose*

Recent in vitro assays that quantify the time necessary to eliminate the parasites (time-to-kill assays) showed that nitroheterocyclic compounds such as BNZ are dose- dependent [[23](#_bookmark3)]. In fact, treatment schemes with a higher- than-standard dose of BNZ (400 mg daily) with the same duration already have been used (STOP-CHAGAS [A Study of the Use of Oral Posaconazole in the Treatment of Asymptomatic Chronic Chagas Disease] study), without observing a higher proportion of side effects [[24](#_bookmark3)]. Further- more, another study of 54 patients treated with BNZ tried to establish a correlation between the serum concentra- tions of the drug and the appearance of AEs. Fifty-three patients (98%) experienced at least one AE during follow- up, but no relationship was found between the drug serum concentration and the occurrence of AEs [[25](#_bookmark3)].

*Shorter regimens*

Finally, regarding the duration of treatment, recent stud- ies in animal models have shown that shorter schemes (25% of standard duration) achieve the same cure rate [[26](#_bookmark3)]. This is under assessment in other clinical trials [[27](#_bookmark3), [28](#_bookmark3)], but findings of one study showed an important cure rate in patients who had to abandon the treatment due to severe adverse events (SAEs) [[29](#_bookmark3)].

# Methods/design

The MULTIBENZ study (Evaluation of Different Benzni- dazole Regimens for the Treatment of Chronic Chagas Disease; ClinicalTrials.gov, NCT03191162) is a phase II, superiority, parallel-arm, randomized, double-blind, multicenter international clinical trial assessing the effi- cacy and safety of three different BNZ dose schemes for the treatment of CD in chronic phase. It will be carried out in four different countries: Argentina, Brazil, Colombia, and Spain (protocol version V1/05-12-2016).

Outcomes and endpoints

The primary objective of MULTIBENZ is to evaluate the ef- ficacy of different BNZ regimens at 12 months after randomization in patients with CD in the chronic phase. The primary efficacy outcome is defined as the proportion of patients with sustained parasitic load suppression in periph- eral blood measured by polymerase chain reaction (PCR) during the first 12 months of follow-up after randomization.

The secondary objectives are to evaluate the parasitic kinetics by detecting parasitic DNA measured by PCR in peripheral blood at different time points (weeks 1, 2, 4, and 8 during the treatment period and in the fourth, sixth, and eighth months after the start of treatment), to evaluate the serological response by enzyme-linked im- munosorbent assay (ELISA) methods at the end of follow-up (month 12), to assess the tolerability and safety of the different BNZ regimens, to correlate BNZ

levels with the therapeutic response and AEs, to correl- ate the presence of HLA-B*3505 with the presence of se- vere AEs, and to correlate the different discrete typing units of *T. cruzi* with the therapeutic response.

*Primary endpoints*

The primary endpoint is parasitologic response, defined as maintained negative qualitative PCR results during the 12-month follow-up period. For efficacy assessments, the end of treatment of each treatment arm will be de- fined in accordance with the duration of the treatment regimen. Incidence and severity of AEs and those lead- ing to treatment discontinuation will also be recorded.

*Secondary endpoints*

Secondary endpoints are parasitic clearance at weeks 1, 2, 4, and 8 during the treatment period and at 4, 6, and 8 months during the follow-up period, measured by qualitative PCR. Serological response will be assessed by conventional serology at 12-month follow-up. The pro- portion of HLA-B*3505 carriers among the patients who experience SAEs will be recorded, as will blood concen- trations of BNZ at 15 d and by the end of treatment.

Patient eligibility

Patients aged ≥ 18 years having any combination of at least two positive serologic test results against *T. cruzi* (indirect immunofluorescence, indirect hemagglutination, or ELISA) and not having previously received treatment with BNZ or nifurtimox (either com- pletely or partially) will be eligible. Inclusion and exclu- sion criteria are summarized in Tables [1](#_bookmark0) and [2](#_bookmark0), respectively.

Randomization and follow-up

Patients are randomly assigned to receive BNZ 150 mg/ day for 60 days, 400 mg/day for 15 days, or the standard scheme of 300 mg/day for 60 days. Because this is a double-blind trial, investigators and all sponsor staff will not be aware of the treatment allocation and randomization list until the end of the trial. Double- blinding will be adopted for all trial arms. Patients will be randomized 1:1:1, and randomization will be done via a remote and interactive response system, according to a predefined list. To avoid bias, randomization will be cen- tralized and concealed. The treatment groups will be al- located on day 1 on the basis of a balanced block randomization, taking into account the country. Each patient will be assigned an identification code that will correspond to the trial kit number allocated to the pa- tient. The label will indicate the trial number and the number of the kit, but it will not indicate the treatment designation.

Table 1 Inclusion criteria

Inclusion criteria (patients should meet all criteria)

- Adults ≥ 18 years old
- Have been diagnosed with Chagas disease by two positive serological tests using different antigens
- Have detectable *Trypanosoma cruzi* DNA in peripheral blood through a qualitative interpretation of the polymerase chain reaction technique
- Written informed consent provided
- Weight ≥ 50 kg and ≤ 80 kg
- Ability to comply with all tests and specified protocol visits and have a permanent address
- Patients must be residents of areas free of vector transmission (*Triatoma infestans*), defined by local health programs or by the Pan American Health Organization/World Health Organization definition.
- Women of childbearing age should have a urine or serum negative pregnancy test at the moment of the baseline visit. Breastfeeding should not be allowed, and a barrier method of contraception should be used during the treatment phase.

Table 2 Exclusion criteria

Exclusion criteria

- Having previously received treatment with benznidazole or nifurtimox (either completely or incompletely)
- Signs and/or symptoms of severe cardiac form of Chagas disease (as confirmed by local national guidelines)
- Impossibility to complete the specified protocol follow-up visits
- Acute or chronic health problems that, in the opinion of the principal investigator, may interfere with the evaluation of the efficacy and/or safety related to the drug (for example, acute infections, human immunodeficiency virus infection, liver or kidney disease)
- History of alcohol abuse
- Known hypersensitivity to metronidazole drugs
- Concomitant use or history of use of allopurinol, antimicrobial, antiparasitic, or antifungal agents
- Having laboratory parameters outside the range of normal or that are considered clinically relevant by the responsible physician:

Total leukocyte count must be within the normal range, with an acceptable range of ± 5%.

Total platelet count must be within the normal range up to 550, 000/mm^3^ or 550 × 10^9^/L.

Total bilirubin must be within the normal range.

Transaminase levels must be within the normal range, with an acceptable range of 25% above the upper limit of normal (ULN).

Total creatinine level must be within the normal range, with an acceptable variation of 10% above the ULN.

Alkaline phosphatase level must be within the normal range up to

< 2.5× ULN.

Gamma glutamyl transferase level must be within the normal range up to 2× ULN.

Fasting glucose must be within the normal range.

Scheduled follow-up visits will occur at 7, 15, 28, and 60 days and up to 4, 6, 8, and 12 months after initiation of treatment. More information is provided in Fig. [1](#_bookmark1).

Sample size and data analysis

For the sample size calculation, we considered a superiority design for two-sample comparison of propor- tions.. we hypothesize a reduction of 50% of the total number of patients who either have a positive PCR result during follow-up or have to discontinue the treatment due to AEs, has been taken into account as a hypothesis. It is estimated that in the standard arm of treatment, 40% of patients will be evaluated as treatment failures according to the intention-to- treat principle.

Given the expected proportions in every group and given that we plan two pairwise comparisons for a power of 80% and a type I error of 0.05, the total number of patients that should be included in the study is 240, which will comprise 60 participants per country.

The categorical data will be presented as absolute numbers and proportions, and the continuous variables will be expressed as means and standard deviations when normal distribution is demonstrated (using the Kolmogorov-Smirnov test) or as medians and interquar- tile ranges when it is not.

For comparison of the distribution of categorical vari- ables, the χ^2^ test or Fisher’s exact test will be used, and the Mann-Whitney *U* test or Student’s *t* test will be used for continuous variables, respectively, depending on the presence or not of normal distribution.

A comparative analysis of the main clinicoepidemiologi- cal variable among the three groups will be carried out. The primary efficacy analysis will be the comparison of the proportion of patients with sustained parasitologic clearance of each treatment arm compared with the stand- ard arm of treatment. The time until the first positive PCR result for *T. cruzi* will be evaluated by Kaplan-Meier sur- vival analysis with the log-rank test for significance.

The proportion of patients with SAEs and/or AEs leading to treatment discontinuation will be described per trial arm and by System Organ Class (using pre- ferred terms defined by MedDRA 13.1), according to the National Cancer Institute’s Common Terminology Cri- teria for Adverse Events (version 4.03). Incidence rate and 95% confidence interval will be presented per trial arm for SAEs and AEs per category, along with the most frequent AEs.

Safety laboratory parameters (hematology and bio- chemistry) will also be described individually per trial arm, showing the proportion of patients by degree of


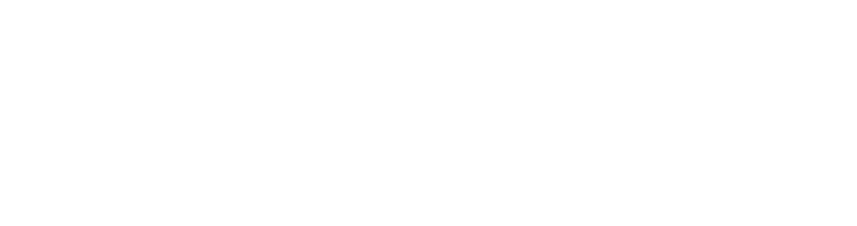

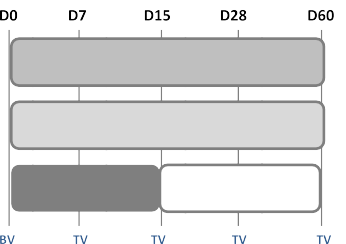

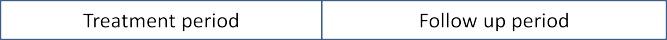

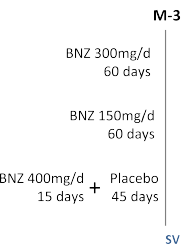


Fig. 1 Clinical trial design. *BNZ* Benznidazole, *BV* Baseline visit, *EoF* End of follow-up, *FV* Follow-up visit, *SV* Screening visit, *TV* Treatment visit

elevation relative to upper limit of normal and baseline values, as well as blood level changes over time.

Safety data will be correlated to efficacy and treatment compliance data and to PK parameters. Differences in the rate of absorption in serology will be estimated with *t* test pairs or the Wilcoxon test, depending on their dis- tribution. All safety analyses will be carried out on all patients treated, understood as all patients who receive at least one dose of treatment. Finally, the sensitivity, specificity, and positive and negative predictive values of the presence of HLA-B*3505 and their relationship with the occurrence of SAEs will be calculated.

The efficacy analysis will be carried out according to the intention-to-treat principle. The group of patients analyzed will be all randomized patients in each of the treatment branches. Cases lost during follow-up and dropouts will be considered treatment failures.In addition, a per-protocol analysis will be defined as all patients receiving randomized treatment who meet the main criteria, have not perman- ently left the administration of the treatment, and have no other protocol deviation. Patients lost to follow-up will be excluded unless they present a positive PCR result, in which case, they will be included in the analysis. The results will be analyzed using IBM SPSS Statistics version 19.0 software (IBM Corp., Armonk, NY, USA).

Laboratory procedures

*Serology*

Two different anti–*T. cruzi* serologic tests based on dif- ferent antigens were used for assessing patient eligibility. To avoid interlaboratory variability, serum samples col- lected at the times indicated in the protocol will be sent at the end of the study to a centralized laboratory that will process them using two techniques in parallel: Architect Chagas (Abbott Laboratories, Wiesbaden, Germany) and ORTHO *Trypanosoma cruzi* ELISA Test System (Ortho Clinical Diagnostics, Raritan, NJ, USA). In order to give greater robustness to the results, sam- ples from external quality control sent by the National Program of Quality Control of Brazil will be included.

*PCR*

Laboratories included in the project will carry out the same reverse transcriptase (RT)-PCR protocol following the in- structions included in the laboratory manual agreed among all of them. To carry out this technique, 5 ml of whole blood will be collected and mixed with 5 ml of guanidine hydro- chloride 6 M–ethylenediaminetetraacetic acid 0.2 M for a minimum of 72 h at room temperature. Three DNA extrac- tions will be performed using the manual column method (High Pure PCR Template Preparation Kit; Roche Diagnos- tics, Mannheim, Germany), except in Spanish centers (Vall d´Hebron University Hospital and Ramón y Cajal University Hospital) where DNA extraction will also be done in tripli- cate using an automated extraction method (NucliSens easy- MAG, bioMèrieux, Marcy l’Etoile, France).The consensual PCR protocol consists of a real-time multiple PCR (Duffy et al. 2013) [[30](#_bookmark3)] that allows the amplification of a *T. cruzi* sat- ellite DNA region and a linearized recombinant plasmid used as an internal amplification control. The RT-PCR will be carried out in duplicate from each of the extractions. At least one amplification of the six performed with an amplifi- cation cycle (cycle threshold) of *T. cruzi* below 40 and a cor- rect value of Internal Amplification Control (IAC) will be interpreted as positive. To be correct, the values of the IAC must meet Tukey’s criteria.

To assess the homogeneity of the results obtained by the different laboratories, a harmonization panel consisting of ten tubes containing blood with uninfected guanidine and infected with 1, 10, and 100 parasitic equivalents per milliliter of *T. cruzi* strains TcV and TcVI was processed. The samples were processed blindly by the different laboratories, and the results were evaluated by an external center in charge of providing the panel and analyzing the results (Instituto de Investigaciones en Ingeniería Genética y Biología Molecular, Buenos Aires, Argentina). On the other hand, and following the same work scheme, four external quality control panels will be analyzed during the study period.

*HLA typing*

The typing of HLA-B alleles is carried out from the dried blood samples on paper (dried blood spots [DBS]).

For this purpose, DNA is extracted using DNA Elution Solution reagent (catalog no. 159994; Qiagen, Carpin- teria, CA, USA), and the concentration and quality are evaluated by measuring the absorbance at 260 and 280 nm using the Colibri microvolume spectrometer (Titer- tek-Berthold, Pforzheim, Germany). The characterization of the HLA-B alleles is carried out by PCR sequence- specific oligonucleotide (SSO) (Luminex Corp., Austin, TX, USA), following the instructions of the Lifecodes HLA typing kit (Immucor; Diagnóstica Longwood, Zara- goza, Spain). Briefly, the PCR-SSO/Luminex consists of amplification with biotinylated primers of the most poly- morphic regions of the HLA-B gene, followed by hybridization of the amplified product with specific probes for each allele located on the surface of Luminex microspheres and revealed with conjugated streptavidin with phycoerythrin. Finally, it is analyzed using an xMAP100 fluoroanalyzer (Luminex Corp.).

*BNZ serum concentration*

Quantification of BNZ is done from dried blood samples on paper (DBS). The quantification is performed by li- quid chromatography (ACQUITY ultra performance li- quid chromatography high-strength silica T3 C18, 2.1 × 50 mm; Waters, Milford, MA, USA) coupled to triple- quadrupole mass spectrophotometry (Xevo TQ; Waters).

Study organization

The MULTIBENZ study network includes four countries and seven centers, with the Spanish coordinating center located at Vall d’Hebron University Hospital in Barce- lona, Spain. The study contemplates a total of three stages, which are outlined below.

1. Recruitment sites

Countries involved in the recruitment will be Spain (University Hospital Vall d’Hebron, Barcelona; University Hospital Ramón y Cajal, Madrid), Argentina (Instituto Nacional de Parasitología Dr. Mario Fatala Chaben, Buenos Aires; Instituto de Cardiología Juana Francisca Cabral, Corrientes), Brazil (Centro de Pesquisas René Rachou – Fundação Oswaldo Cruz, Belo Horizonte; Hospital Univer- sitário Clemente de Faria, Montes Claros), and Colombia (Fundación Cardioinfantil – Instituto de Cardiología).

1. Selection phase

Patients with CD in the chronic phase who come to the study centers will be evaluated in order to assess if they meet the inclusion criteria. For those who agree to participate and sign the informed consent, detection of parasitic DNA by PCR in peripheral blood will be per- formed. Patients with a PCR result negative for *T. cruzi*

will be withdrawn from the study. The screening proced- ure must occur up to 90 days or less before the initiation of treatment. Serology and DNA determination by PCR will be accepted as valid and will not need to be repeated if a posi- tive result was obtained in a previous period of 3 months.

All patients will undergo a clinical history and physical examination. Peripheral blood extraction will be per- formed for analysis: hemogram, biochemistry assays, and HLA study, and a negative pregnancy test (either in urine or blood) is mandatory in case of women in child- bearing age. The evaluation of the visceral involvement of CD will be completed with a chest x-ray and electro- cardiogram. The performance of other complementary tests will be carried out according to the clinical investi- gator’s decision, but they will not be considered neces- sary for the inclusion of the patient.

1. Treatment phase

In this phase, the patient will be randomized to one of the three arms of treatment, whose duration will be 60 days in- dependently of the arm assigned. Initially, a baseline visit will be performed in which the patient will be trained on how to take the drug and identify AEs. After that, a total of four scheduled visits will occur during the treatment period, at 7, 15, 28, and 60 days after treatment initiation. A summary of these visits is presented in Table [3](#_bookmark2).

During this phase, any patient may consult spontan- eously for the eventual occurrence of any AE. The deci- sion to interrupt the treatment will be according to the discretion of the clinical researcher treating the patient, taking into account the severity, intensity, and extent of these AEs. In addition, the patient may decide unilat- erally to suspend or not the medication at any time dur- ing the treatment period.

1. Follow-up phase

Once the patient has taken the last dose of treatment, which may be at the end of the therapeutic scheme (day 60), when a severe AE that obliges the patient to sus- pend the medication occurs, or when the patient unilat- erally decides to suspend the medication, this phase will begin, and it will last up to 12 months after randomization. The visit schedule of this phase is pre- sented in Table [3](#_bookmark2).

SAEs and unblinding: care of patients with AEs

All AEs and other study outcomes in the randomization, treatment, and follow-up periods will have to be re- ported. In case of mild or moderate AEs, the administra- tion of BNZ will be suspended temporarily according to the clinical researcher’s decision. Symptomatic treatment

Table 3 Visit schedule

|  | Selection visit (from − 3 months | Baseline visit | Treatment visit | Treatment visit | Treatment visit | End of treatment visit | Side effect visit | Follow-up visit (months 4, 6, | Final visit |
| --- | --- | --- | --- | --- | --- | --- | --- | --- | --- |
|  | to day 0) | (day 0) | (day 7 ± 2) | (day 15 ±  3) | (day 28 ±  4) | (day 60 ± 7) | (not scheduled) | and 8) | (month 12) |
| Informed consent | x |  |  |  |  |  |  |  |  |
| Demographic data | x |  |  |  |  |  |  |  |  |
| Pathological background | x |  |  |  |  |  |  |  |  |
| Toxic habits | x |  |  |  |  |  |  |  |  |
| Chagas disease history | x |  |  |  |  |  |  |  |  |
| Concomitant medication | x | x | x | x | x | x | x | x | x |
| Directed anamnesis | x | x | x | x | x | x | x | x | x |
| Physical examination | x | x | x | x | x | x | x | x | x |
| Pregnancy test |  | x |  |  |  |  |  |  |  |
| Hematology | x |  | x | x | x | x | x |  |  |
| Biochemistry | x |  | x | x | x | x | x |  |  |
| HLA | x |  |  |  |  |  |  |  |  |
| Chest x-ray | x |  |  |  |  |  |  |  |  |
| EKG | x |  |  |  |  |  |  |  |  |
| PK |  |  |  | x |  | x | x |  |  |
| Serology | x |  |  |  |  |  |  |  | x |
| PCR | x |  | x | x | x | x |  | x | x |
| Other complementary tests | x |  |  |  |  |  |  |  |  |
| Adverse events |  | x | x | x | x | x | x |  |  |
| Randomization | x |  |  |  |  |  |  |  |  |
| Study medication |  | x | x | x | x | x |  |  |  |

*Abbreviations: EKG* Electrocardiogram, *HLA* Human leukocyte antigen, *PCR* Polymerase chain reaction, *PK* Pharmacokinetics

will be provided to the patient according to the treating physician.

It will be considered that a patient has finished the study when he or she has completed the treatment and follow-up phase (per-protocol principle). The study may be interrupted in case of voluntary decision of the pa- tient at any time if an AE forces interruption of the treatment, if significant protocol violations occur, or if the treating physician deems it would benefit the patient (intention-to-treat principle).

Ethics and patient confidentiality

The protocol has been approved by national regulatory agencies (in accordance with the ethical standards laid down in the Declaration of Helsinki as revised in 2013), by the institutional review boards for clinical research of all participating institutions, and by the national ethics review committees of the countries involved in the study (when applicable). All patients provide written informed consent,

whereby the researcher will explain to each patient the na- ture of the study; its purposes, procedures, expected dur- ation, and the potential risks and benefits related to participating; and any inconvenience that may occur.

# Discussion

CD continues to be an infectious disease with one of the highest disease burdens worldwide. Despite the modest cure rates in adult chronic patients and its safety profile, BNZ remains the best treatment option against the dis- ease due to the lack of therapeutic alternatives [[7](#_bookmark3)]. Con- ventional diagnostic methods for establishing cure rates in chronic CD have marked inherent limitations; how- ever, the use of more sensitive methods for parasite de- tection, such as PCR, could provide a suitable tool for follow-up assessment of treatment in patients with CD, because detectable *T. cruzi* DNA in peripheral blood samples after treatment end is considered a therapeutic failure [[31](#_bookmark4)].

The currently used dose of BNZ is based on nonran- domized studies that were carried out more than 50 years ago, and they have never been analyzed in clinical trials. Moreover, there are no data regarding the rela- tionship between dose and efficacy, because the great majority of published experience (clinical trials and ob- servational studies) has been with the standard dose.

Recent studies have brought to light interesting results that point out different possibilities of treatment schemes. On the basis of two PK studies, authors sug- gested that current treatment regimens could be over- dosed [[19](#_bookmark3), [20](#_bookmark3)]. One them extrapolated the idea according to the lower concentration observed in treated children that finally achieved cure. The other based the conclusion on dosage regimen simulation under steady- state conditions comparing the estimated concentration with the optimal therapeutic accepted range. This hy- pothesis has also been observed preclinically using an in vivo model under the framework of the Berenice Pro- ject (Benznidazol and Triazol Research Group for Nano- medicine and Innovation on Chagas Disease). Results obtained from the murine model led researchers to con- clude that a dose of 40% of the total of the version used as standard is comparable in efficacy.

At the same time, there is evidence of the opposite. According to mechanism of action of BNZ, it seems that the efficacy of the drug (and all nitroderivative drugs) is concentration-dependent. To include an arm with higher dose in a clinical trial could pose an increased risk of toxicity. The clinical experience obtained through clin- ical trials with higher doses of BNZ (400 mg/day) led to the belief that higher dose is not associated with higher frequency of AEs [[23](#_bookmark3), [24](#_bookmark3)].

In addition, the mechanisms underlying BNZ toxicity are still not well understood. The production of several metabolites in the enzymatic reduction of the drug, their accumulation, and their interactions with cellular con- stituents could be the main reasons for producing these AEs [[32](#_bookmark4), [33](#_bookmark4)]. Some AEs of the drug have a certain tem- poral pattern. For example, dermatological and digestive manifestations usually tend to occur around day 10 of treatment, while neurological events and arthritis appear after day 40 of treatment, probably because they are re- lated to the cumulative total dose and not to the serum concentration [[9](#_bookmark3)]. Age and female sex have also been considered as classic risk factor for AEs. However, there remains an important lack of knowledge about the mechanisms of toxicity [[9](#_bookmark3), [10](#_bookmark3)].

According to the duration of the treatment, two ap- proaches were considered. Taking into account the cure rates of patients with CD treated with shorter courses of BNZ (those who had to interrupt the treatment because of AEs), a shortened regimen has been incorporated. Finally, re- searchers explored the possibility of including a new arm

with prolonged exposure. Unfortunately, due to the lack of clinical published experience and the potential risk of go- nadal toxicity and its effect over the pituitary–testicular axis, that treatment arm was discarded [[34](#_bookmark4), [35](#_bookmark4)].

Another aspect that will be assessed is the genetic variabil- ity of the parasite over the treatment response. The great majority of randomized clinical trials have been conducted in Argentinean or Bolivian patients, and scarce information is available in other geographical regions. In any case, the few existing data suggest an important effect of parasitic di- versity in the treatment efficacy and response [[17](#_bookmark3), [36](#_bookmark4)].

Therefore, this clinical trial will evaluate the efficacy and safety of different dosing regimens of BNZ com- pared with the standard treatment scheme. Experimental dosing regimens have been chosen on the basis of evi- dence derived from previous studies, in which it has been found that shorter-duration schemes and/or lower dose with the same duration could achieve the same cure rate. Moreover, higher doses could be used without entailing a higher rate of AEs. The results of this clinical trial will help to better identify the most adequate BNZ regimen in terms of efficacy and safety for the treatment of CD in the chronic phase.

The MULTIBENZ study is included within the Bere- nice Project, founded by the European Commission and initiated in September 2012. The aim of the Berenice Project is to provide a new and cost-effective solution to treat patients with CD in chronic phase and to develop new drug formulations with trypanocidal activity. Its main objective is to obtain a more effective, better toler- ated, and cheaper treatment to cure CD. The results ob- tained in the Berenice Project will upgrade European competitiveness through the transformation of research in the field of neglected infectious diseases.

# Trial status

Recruitment was started in April 2018, and it is estimated to be completed in all countries by August 2020. Protocol ver- sion V1/ 05-12-2016.

Acknowledgements

Authors from the Drugs for Neglected Diseases initiative (DNDi) thank its donors, public and private, who have provided funding to DNDi since its inception in 2003. A full list of DNDi donors can be found at <https://www.dndi.org/donate/donors/>.

Authors’ contributions

DMM, PBN, FS, and ASM wrote the original draft of the manuscript and analyzed and interpreted data. MLF, MB, ALPR, AMBP, SE, RCO, JCV, and SSE wrote, reviewed, and edited the manuscript. ES analyzed data. IM wrote the original draft of the manuscript, analyzed and interpreted data, and designed the work. All authors read and approved the final manuscript.

Funding

This clinical trial is supported by the European Berenice Project, a collaborative project funded under the European Community’s 7th Framework Program (grant agreement HEALTH-305937). The funder had no role in the clinical trial design; in the collection, analysis, and interpretation of data; in the writing of the report; or in the decision to submit the article for publication.

Availability of data and materials

The datasets generated and analyzed during the study will be available from the corresponding author on reasonable request.

Ethics approval and consent to participate

The protocol has been approved by national regulatory agencies (in accordance with the ethical standards laid down in the Declaration of Helsinki as revised in 2013), by the institutional boards for clinical research of all participating institutions, and by the national ethics review committees of the countries involved in the study (when applicable). Referral ethical board: Vall d’Hebron University Hospital Ethical Committee on Clinical Research with Drugs (EUDRACT no. 2016-003789-21). All patients provide written informed consent, whereby the researcher will explain to each patient the nature of the study; its purposes, procedures, and expected duration; the potential risks and benefits related to participating; and any inconvenience that may occur.

Consent for publication

Not applicable.

Competing interests

The authors declare that they have no competing interests.

Author details

^1^Infectious Diseases Department, Vall d’Hebron University Hospital, PROSICS Barcelona, Universitat Autònoma de Barcelona, P° Vall d’Hebron 119, Edifici Mediterrània, VHIR, 08035 Barcelona, Spain. ^2^Departamento de Clínica, Patología y Tratamiento, Instituto Nacional de Parasitología Dr. Mario Fatala Chaben, Ministerio de Salud y Desarrollo Social, Buenos Aires, Argentina.

^3^Microbiology Department, Vall d’Hebron University Hospital, PROSICS Barcelona, Universitat Autònoma de Barcelona, Barcelona, Spain. ^4^Instituto de Cardiología de Corrientes Juana Francisca Cabral (Argentina), Corrientes, Argentina. ^5^Programa de Pós-graduação Infectologia e Medicina Tropical, Faculdade de Medicina da Universidade Federal de Minas Gerais, Belo Horizonte, Minas Gerais, Brazil. ^6^Laboratory of Health Science, Postgraduate Program in Health Sciences, Universidade Estadual de Montes Claros (Unimontes), Montes Claros, MG, Brazil. ^7^Programa de Pós-graduação em Patologia, Departamento de Propedêutica Complementar, Faculdade de Medicina da Universidade Federal de Minas Gerais, Belo Horizonte, Brazil.

^8^Faculdade de Medicina da Universidade José do Rosário Vellano, Belo Horizonte, Brazil. ^9^René Rachou Institute, Oswaldo Cruz Foundation, Belo Horizonte, Brazil. ^10^Faculty of Health Sciences, Universidad Autónoma de Bucaramanga and Research Department, Bucaramanga, Colombia.

^11^Fundación Cardioinfantil - Instituto de Cardiología, Bogotá, Colombia. ^12^Chagas Clinical Program, Drugs for Neglected Disease initiative (DNDi), Geneva, Switzerland. ^13^Epidemiology and Public Health Research Center, CONICET, Buenos Aires, Argentina.

Received: 2 October 2019 Accepted: 3 March 2020


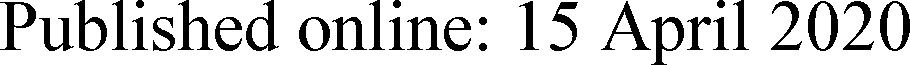


References

1. WHO. Chagas disease in Latin America: an epidemiological update based on 2010 estimates. Wkly Epidemiol Rec. 2015;90(6):33–43..
2. Molina I, Salvador F, Sánchez-Montalvá A. Update Chagas disease [in Spanish]. Enferm Infecc Microbiol Clin. 2016;34(2):132–8.
3. Pérez-Molina JA, Molina I. Chagas disease. Lancet. 2018;391(10115):82–94.
4. Noya BA, Pérez-Chacón G, Díaz-Bello Z, Dickson S, Muñoz-Calderón A, Hernández C, et al. Description of an oral Chagas disease outbreak in Venezuela, including a vertically transmitted case. Mem Inst Oswaldo Cruz. 2017;112(8):569–71.
5. Sánchez-Montalvá A, Salvador F, Rodríguez-Palomares J, Sulleiro E, Sao- Avilés A, Roure S, et al. Chagas cardiomyopathy: usefulness of EKG and echocardiogram in a non-endemic country. PLoS One. 2016;11(6):e0157597.
6. Machado FS, Jelicks LA, Kirchhoff LV, Shirani J, Nagajyothi F, Mukherjee S, et al. Chagas heart disease: report on recent developments. Cardiol Rev. 2012;20(2):53–65.
7. Sales Junior PA, Molina I, Fonseca Murta SM, Sánchez-Montalvá A, Salvador F, Corrêa-Oliveira R, et al. Experimental and clinical treatment of Chagas disease: a review. Am J Trop Med Hyg. 2017;97(5):1289–303.
8. Bern C. Antitrypanosomal therapy for chronic Chagas’ disease. N Engl J Med. 2011;364(26):2527–34.
9. Molina I, Salvador F, Sánchez-Montalvá A, Treviño B, Serre N, Sao Avilés A. Toxic profile of benznidazole in patients with chronic Chagas disease: risk factors and comparison of the product from two different manufacturers. Antimicrob Agents Chemother. 2015;59(10):6125–31.
10. Hasslocher-Moreno AM, do Brasil PEAA, de Sousa AS, Xavier SS, Chambela MC, Sperandio da Silva GM. Safety of benznidazole use in the treatment of chronic Chagas’ disease. J Antimicrob Chemother. 2012;67(5):1261–6.
11. Salvador F, Sánchez-Montalvá A, Martínez-Gallo M, Sala-Cunill A, Viñas L, García-Prat M, et al. Evaluation of cytokine profile and HLA association in benznidazole related cutaneous reactions in patients with Chagas disease. Clin Infect Dis. 2015;61(11):1688–94.
12. Coura JR, Borges-Pereira J. Chronic phase of Chagas disease: why should it be treated? A comprehensive review. Mem Inst Oswaldo Cruz. 2011;106(6):641–5.
13. Sguassero Y, Roberts KN, Harvey GB, Comandé D, Ciapponi A, Cuesta CB, et al. Course of serological tests in treated subjects with chronic *Trypanosoma cruzi* infection: a systematic review and meta-analysis of individual participant data. Int J Infect Dis. 2018;73:93–101.
14. Pan American Health Organization (PAHO). Guidelines for the diagnosis and treatment of Chagas disease. Washington, DC: PAHO; 2018. [http://iris.paho.](http://iris.paho.org/xmlui/handle/123456789/49653) [org/xmlui/handle/123456789/49653](http://iris.paho.org/xmlui/handle/123456789/49653).
15. Fabbro DL, Danesi E, Olivera V, Codebó MO, Denner S, Heredia C, et al. Trypanocide treatment of women infected with *Trypanosoma cruzi* and its effect on preventing congenital Chagas. PLoS Negl Trop Dis. 2014;8(11):e3312.
16. Perez-Molina JA, Perez-Ayala A, Moreno S, Fernandez-Gonzalez MC, Zamora J, Lopez-Velez R. Use of benznidazole to treat chronic Chagas’ disease: a systematic review with a meta-analysis. J Antimicrob Chemother. 2009;64:1139–47.
17. Morillo CA, Marin-Neto JA, Avezum A, et al. Randomized trial of benznidazole for chronic Chagas’ cardiomyopathy. N Engl J Med. 2015;373:1295–306.
18. Richle RW, Raaflaub J. Difference of effective antitrypanosomal dosages of Benznidazole in mice and man. Chemotherapeutic and pharmacokinetic results. Acta Trop. 1980;37(3):257–61..
19. Altcheh J, Moscatelli G, Mastrantonio G, Moroni S, Giglio N, Marson ME,

et al. Population pharmacokinetic study of benznidazole in pediatric Chagas disease suggests efficacy despite lower plasma concentrations than in adults. PLoS Negl Trop Dis. 2014;8(5):e2907.

1. Soy D, Aldasoro E, Guerrero L, Posada E, Serret N, Mejía T, et al. Population pharmacokinetics of benznidazole in adult patients with Chagas disease. Antimicrob Agents Chemother. 2015;59(6):3342–9.
2. Raaflaub J, Ziegler WH. Single-dose pharmacokinetics of the trypanosomicide benznidazole in man. Arzneimittelforschung. 1979;29:1611–4.
3. Raaflaub J. Multiple-dose kinetics of the trypanosomicide benznidazole in man. Arzneimittelforschung. 1980;30:2192–4.
4. Moraes CB, Giardini MA, Kim H, Franco CH, Araujo-Junior AM, Schenkman S, et al. Nitroheterocyclic compounds are more efficacious than CYP51 inhibitors against *Trypanosoma cruzi*: implications for Chagas disease drug discovery and development. Sci Rep. 2014;4:4703.
5. Morillo CA, Waskin H, Sosa-Estani S, Del Carmen Bangher M, Cuneo C, Milesi

R. Benznidazole and posaconazole in eliminating parasites in asymptomatic

*T. Cruzi* carriers: the STOP-CHAGAS trial. J Am Coll Cardiol. 2017;69(8):939–47.

1. Pinazo MJ, Guerrero L, Posada E, Rodríguez E, Soy D, Gascon J. Benznidazole- related adverse drug reactions and their relationship to serum drug concentrations in patients with chronic Chagas disease. Antimicrob Agents Chemother. 2013;57(1):390–5.
2. Francisco AF, Lewis MD, Jayawardhana S, Taylor MC, Chatelain E, Kelly JM. Limited ability of Posaconazole to cure both acute and chronic *Trypanosoma cruzi* infections revealed by highly sensitive in vivo imaging. Antimicrob Agents Chemother. 2015;59(8):4653–61.
3. BENDITA (Benznidazole New Doses Improved Treatment and Associations) study. ClinicalTrials.gov identifier: NCT03378661. [https://clinicaltrials.gov/ct2/](https://clinicaltrials.gov/ct2/show/NCT03378661) [show/NCT03378661](https://clinicaltrials.gov/ct2/show/NCT03378661). Accessed 11 Mar 2020.
4. Álvarez MG, Hernández Y, Bertocchi G, Fernández M, Lococo B, Ramírez JC, et al. New scheme of intermittent benznidazole administration in patients chronically infected with *Trypanosoma cruzi*: a pilot short-term follow-up study with adult patients. Antimicrob Agents Chemother. 2015;60(2):833–7.
5. Alvarez MG, Vigliano C, Lococo B, Petti M, Bertocchi G, Viotti R. Seronegative conversion after incomplete benznidazole treatment in chronic Chagas disease. Trans R Soc Trop Med Hyg. 2012;106:636–8.
6. Duffy T, Cura CI, Ramirez JC, Abate T, Cayo NM, Parrado R, Bello ZD, Velazquez E, Muñoz-Calderon A, Juiz NA, Basile J, Garcia L, Riarte A, Nasser JR, Ocampo SB, Yadon ZE, Torrico F, de Noya BA, Ribeiro I, Schijman AG. Analytical performance of a multiplex Real-Time PCR assay using TaqMan

probes for quantification of Trypanosoma cruzi satellite DNA in blood samples. PLoS Negl Trop Dis. 2013;7(1):e2000.

1. Gomes ML, Galvao LM, Macedo AM, et al. CD diagnosis: comparative analysis of parasitologic, molecular, and serologic methods. Am J Trop Med Hyg. 1999;60:205–10.
2. Viotti R, Vigliano C, Lococo B, Alvarez MG, Petti M, Bertocchi G, Armenti A. Side effects of benznidazole as treatment in chronic Chagas disease: fears and realities. Expert Rev Anti Infect Ther. 2009;7:157–63.
3. Castro JA, de Mecca MM, Bartel LC. Toxic side effects of drugs used to treat Chagas’ disease (American trypanosomiasis). Hum Exp Toxicol. 2006;25:471–9.
4. Favaretto AL, Antunes-Rodrigues J, Vieira CL, Lamano-Carvalho TL. Pituitary-testicular axis in benznidazole-treated rats. Braz J Med Biol Res. 1990;23(8):719–22.
5. Vieira CL, Lamano-Carvalho TL, Favaretto AL, Valença MM, Antunes- Rodrigues J, Barreira AA. Testes alterations in pubertal benznidazole-treated rats. Braz J Med Biol Res. 1989;22(6):695–8.
6. Yun O, Lima MA, Ellman T, Chambi W, Castillo S, Flevaud L, et al. Feasibility, drug safety, and effectiveness of etiological treatment programs for Chagas disease in Honduras, Guatemala, and Bolivia: 10-year experience of Médecins Sans Frontières. PLoS Negl Trop Dis. 2009;3(7):e488.

# Publisher’s Note

Springer Nature remains neutral with regard to jurisdictional claims in published maps and institutional affiliations.


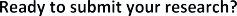

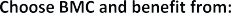

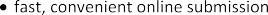

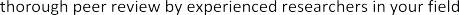

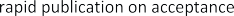

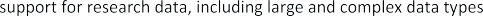

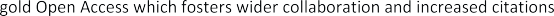

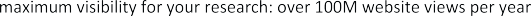

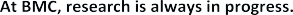

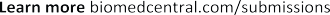

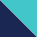

Supplement: Supplementary file 1 — Additional file 1. [file 13063_2023_7659_MOESM1_ESM.docx]
